# Разработка прогностических клинико-генетических моделей риска развития первичного остеопороза с использованием нейросетевого обучения

**DOI:** 10.14341/probl13421

**Published:** 2024-01-24

**Authors:** Б. И. Ялаев, А. В. Новиков, И. Р. Минниахметов, Р. И. Хусаинова

**Affiliations:** Национальный медицинский исследовательский центр эндокринологии; Национальный медицинский исследовательский центр эндокринологии; Национальный медицинский исследовательский центр эндокринологии; Национальный медицинский исследовательский центр эндокринологии; Институт биохимии и генетики Уфимского федерального исследовательского центра РАН

**Keywords:** остеопороз, машинное обучение, нейросеть, генетика

## Abstract

ОБОСНОВАНИЕ. Остеопороз является распространенным возраст-зависимым заболеванием с инвалидизирующими последствиями, ранняя диагностика которого осложнена ввиду длительного и скрытого течения, что зачастую приводит к постановке диагноза только после случая перелома. В этой связи большие надежды возлагаются на передовые разработки в области технологий машинного обучения, направленные на прогнозирование остеопороза на ранней стадии развития, в том числе с применением больших массивов данных, содержащих информацию о генетических и клинических предикторах заболевания. Тем не менее включение ДНК-маркеров в модели прогнозирования сопряжено с рядом трудностей, связанных со сложной полигенной и гетерогенной природой заболевания. На данный момент прогностическая сила нейросетевых моделей недостаточна для их внедрения в современные протоколы диагностики остеопороза. Исследования в этой области единичны, но являются широко востребованными, поскольку их результаты представляют огромную значимость для профилактической медицины. Это приводит к необходимости поиска наиболее эффективных подходов машинного обучения и оптимизации отбора генетических маркеров в качестве входных параметров в нейросетевые модели.ЦЕЛЬ: оценить эффективность машинного обучения и нейросетевого анализа для разработки прогностических моделей риска развития остеопороза на основе клинических предикторов и ДНК-маркеров остеопоротических переломов.МАТЕРИАЛЫ И МЕТОДЫ. Прогностические модели обучены с использованием данных генотипов по 152 полиморфных ДНК-локусов и клинических параметров 701 женщины и 501 мужчины из Волго-Уральского региона России. В качестве входных параметров в модели включены антропометрические показатели, данные о гендерной принадлежности, уровне минеральной плотности костной ткани (МПКТ), а также результаты генотипирования полиморфных локусов генов-кандидатов и локусов репликации полногеномного поиска ассоциаций (GWAS) консорциума GEFOS.РЕЗУЛЬТАТЫ. Установлено, что наиболее высокой эффективности (AUC=0,81 для мужчин и AUC=0,82 для женщин на независимом датасете (англ. dataset — обработанный и структурированный массив данных) достигает модель прогнозирования низкого уровня минеральной плотности костной ткани, в которую вошли 6 полиморфных вариантов гена остеопротегерина (OPG) (rs2073618, rs2073617, rs7844539, rs3102735, rs3134069) и 5 полиморфных вариантов сайтов связывания микроРНК в мРНК таргетных генов, участвующих в костном метаболизме (COL11A1 — rs1031820, FGF2 — rs6854081, miR-146 — rs2910164, ZNF239 — rs10793442, SPARC — rs1054204 и VDR — rs11540149).ЗАКЛЮЧЕНИЕ. Результаты подтверждают перспективность применения машинного обучения для прогнозирования риска развития остеопороза на доклинической стадии заболевания на основе анализа клинических и генетических факторов.

## Abstract (en)

BACKGROUND: Osteoporosis is a common age-related disease with disabling consequences, the early diagnosis of which is difficult due to its long and hidden course, which often leads to diagnosis only after a fracture. In this regard, great expectations are placed on advanced developments in machine learning technologies aimed at predicting osteoporosis at an early stage of development, including the use of large data sets containing information on genetic and clinical predictors of the disease. Nevertheless, the inclusion of DNA markers in prediction models is fraught with a number of difficulties due to the complex polygenic and heterogeneous nature of the disease. Currently, the predictive power of neural network models is insufficient for their incorporation into modern osteoporosis diagnostic protocols. Studies in this area are sporadic, but are widely demanded, as their results are of great importance for preventive medicine. This leads to the need to search for the most effective machine learning approaches and optimise the selection of genetic markers as input parameters to neural network models.AIM: to evaluate the effectiveness of machine learning and neural network analysis to develop predictive risk models for osteoporosis based on clinical predictors and genetic markers of osteoporetic fractures.MATERIALS AND METHODS: The predictive models were trained using a database of genotyping and clinical characteristics of 701 women and 501 men living in the Volga-Ural region of Russia. Anthropometric parameters, data on gender, bone mineral density level, and the results of genotyping of 152 polymorphic loci of candidate genes and replication loci of the GEFOS consortium’s full genome-wide association search were included as input parameters.RESULTS: It was found that the model for predicting low bone mineral density, including 6 polymorphic variants of the OPG gene (rs2073618, rs2073617, rs7844539, rs3102735, rs3134069) and 5 polymorphic variants of microRNA binding sites in the mRNA of genes involved in bone metabolism (COL11A1 — rs1031820, FGF2 — rs6854081, miR-146 — rs2910164, ZNF239 — rs10793442, SPARC — rs1054204 and VDR — rs11540149) (AUC=0.81 for men and AUC=0.82 for women).CONCLUSION: The results confirm the promising application of machine learning to predict the risk of osteoporosis at the preclinical stage of the disease based on the analysis of clinical and genetic factors.

## ОБОСНОВАНИЕ

Остеопороз (ОП) является одним из наиболее распространенных многофакторных заболеваний современности, ежегодно приводящий к более 9 млн низкотравматичных переломов [1]. Известно, что остеопорозу подвержено более 1 млрд человек [2]. В России заболевание диагностируется примерно у 34% женщин и 27% мужчин при проведении денсиметрического обследования на независимых или случайных выборках [3–5]. Ранняя диагностика развития ОП осложнена ввиду длительного и скрытого течения заболевания, что приводит к низкой доле выявляемости ОП и отсутствию ранней профилактики переломов. Остеопороз является социально значимым заболеванием с инвалидизирующим течением, и, ввиду этого, необходима разработка способов ранней диагностики, профилактики и лечения.

При остеопорозе резорбция костной ткани преобладает над минерализацией и анаболическими процессами, что приводит к нарушению прочности костей из-за уменьшения минеральной плотности и увеличению риска переломов, даже от незначительных падений или ударов [6]. У подавляющей доли пациентов остеопороз диагностируется только после перелома, это определяется острой нехваткой методов ранней диагностики на скрининговом уровне.

Геномные и мультиомиксные проекты демонстрируют, что остеопороз в значительной мере ассоциирован с наследственными факторами [7]. Известное исследование с проведенным метаанализом результатов полногеномного поиска ассоциаций (GWAS) выявило порядка 56 локусов, ассоциированных с низким уровнем минеральной плотности костной ткани (МПКТ) и 14 локусов, связанных с риском переломов [8]. Но стадия репликация данного GWAS не подтвердила большую часть выявленных маркеров в популяциях России, проведенное на выборках женщин с остеопорозом из Волго-Уральского региона [8].

Тем не менее достоверно установлено, что наследственность определяет до 65% вариабельности уровня МПКТ и около 25% риска переломов [9], но в то же время число ДНК-локусов с высоким рисковым эффектом (OR>3) невелико [10]. В совокупности подобные факторы создают сложности при разработке способов ранней диагностики ОП и профилактики переломов с использованием данных генетического анализа. Таким образом, несмотря на то что исследования генетики остеопороза ведутся активно уже на протяжении 15 лет, потенциал накопленных данных в качестве диагностического инструментария остается нереализованным [11].

С этой точки зрения многообещающими и внушающими перспективы являются технологии машинного обучения и искусственного интеллекта, получающих все более широкое распространение в области клинической и предиктивной медицины. В отличие от рутинных статистических методов и моделей, технологии на базе машинного обучения (МО) и нейросетевого анализа (НА) позволяют обнаруживать скрытые связи и закономерности в изначально разнородных данных. Следует заметить, что модели на основе искусственного интеллекта нашли применение при остеопорозе как в качестве моделирования риска перелома, так и для анализа рентгеновских изображений [12].

## ЦЕЛЬ ИССЛЕДОВАНИЯ

Оценить эффективность машинного обучения и нейросетевого анализа для разработки прогностических моделей риска развития остеопороза на основе клинических предикторов и ДНК-маркеров остеопоротических переломов.

## МАТЕРИАЛЫ И МЕТОДЫ

## Место и время проведения исследования

Место проведения. Исследование проведено в Федеральном государственном бюджетном научном учреждении «НМИЦ эндокринологии» Минздрава России.

Время исследования. В исследование включены пациенты, которые прошли медицинское обследование в период с 2004 по 2011 гг. на базе городских клинических больниц №5, №21 и №22 г. Уфы и Областной клинической больницы №1 из г. Екатеринбурга.

## Изучаемые популяции

В исследовании приняли участие 701 женщина в постменопаузе (средний возраст = 61,95±7,94) и 501 мужчина (средний возраст 62±10,8). Этнический состав женщин: русские — 516, татары — 185. Этнический состав мужчин: русские — 470, татары — 31.

Критерии включения: выборку пациентов составили люди с остеопорозом, в контрольную группу вошли люди без переломов и с нормальным уровнем минеральной плотности костной ткани в возрасте от 60 до 70 лет. Учитывалось наличие остеопоротических переломов в стандартных локализациях (аксиальная часть бедра, поясничный отдел позвоночника) в целом и по отдельности, а также в сочетании с любыми другими переломами скелета.

Критерии исключения: наличие каких-либо семейных групп и родственников на основе анкетирования и данных семейного анамнеза, злоупотребление алкоголем и наркотиками в анамнезе, текущее лечение острых заболеваний, а также наличие хронических заболеваний, которые влияют на метаболизм костной ткани (бронхиальная астма, заболевания щитовидной железы, аутоиммунные заболевания).

## Дизайн исследования

Работа является одноцентровым одномоментным исследованием. Выборка сформирована в соответствии с критериями включения и исключения.

На первом этапе была определена группа мужчин и женщин старше 50 лет, для которых описан клинический анамнез и проведено генотипирование полиморфных ДНК-локусов, включая данные репликации GWAS-исследования консорциума GEFOS/GENOMOS, а также данные предыдущих ген-кандидатных исследований на данной когорте пациентов. Клинические характеристики групп представлены в таблице 1. Характеристики включенных в исследование ДНК-локусов приведены в таблице 2.

**Table:** Таблица 1. Характеристики исследования по фенотипическим подгруппам

Выборка женщин (N=701)
Группы сравнения	N	Возраст, Me±SD	ИМТ, кг/м², Me±SD
С переломами	280	62,16±7,95	27,00±3,60
Без переломов	421	60,14±8,01	27,80±3,81
С низким уровнем МПКТ	324	62,17±7,95	27,04±3,60
С нормальным уровнем МПКТ	172	60,23±7,97	28,10±4,90
С нормальным уровнем МПКТ и без переломов	239	61,76±7,96	28,90±3,70
Выборка мужчин (N=501)
С переломами	145	62,30±10,83	27,54±2,73
Без переломов	356	59,20±9,02	27,59±4,90
С низким уровнем МПКТ	304	62,17±6,90	27,04±3,60
С нормальным уровнем МПКТ	197	61,23±9,01	27,61±4,60
С нормальным уровнем МПКТ и без переломов	168	61,57±6,97	27,82±2,70

**Table:** Таблица 2. Характеристика полиморфных вариантов, включенных в модели обучения

№	ID локуса	№	ID локуса	№	ID локуса	№	ID локуса
1	rs1061947	39	rs6532023	77	rs6231	115	rs5952638
2	rs11540149	40	rs6830890	78	rs3736228	116	rs4492531
3	rs1042673	41	rs1366594	79	rs2887571	117	rs964181
4	rs1054204	42	rs4957742	80	rs11048046	118	rs1514348
5	rs9659030	43	rs17284960	81	rs7953528	119	rs3020314
6	rs1031820	44	rs9466056	82	rs1282108	120	rs1062033
7	rs5854	45	rs11755164	83	rs2016266	121	rs28757190
8	rs2910164	46	rs13204965	84	rs736825	122	rs1801725
9	rs11614913	47	rs4869742	85	rs1053051	123	rs1801197
10	rs2120461	48	rs7751941	86	rs9533090	124	rs182549
11	rs2295294	49	rs7788807	87	rs7326472	125	rs4988235
12	rs7521902	50	rs28425	88	rs1286083	126	rs2036417
13	rs6426749	51	rs10226308	89	rs3748317	127	rs9630182
14	rs12137389	52	rs6959212	90	rs11623869	128	rs7125774
15	rs17482952	53	rs2282930	91	rs2118784	129	rs3102734
16	rs12407028	54	rs4727338	92	rs129333	130	rs2073618
17	rs11809524	55	rs13245690	93	rs9921222	131	rs2073617
18	rs7417366	56	rs3801387	94	rs13336428	132	rs7844539
19	rs479336	57	rs7812088	95	rs4985155	133	rs3102735
20	rs12120297	58	rs1670346	96	rs1564981	134	rs3134069
21	rs13413210	59	rs1405534	97	rs1566045	135	rs198470
22	rs7584262	60	rs7017914	98	rs1048146	136	rs10793442
23	rs4233949	61	rs13272568	99	rs4790881	137	rs10518716
24	rs730402	62	rs2062377	100	rs4792909	138	rs17054320
25	rs17040773	63	rs10756362	101	rs227584	139	rs1712
26	rs1878526	64	rs11788458	102	rs1864325	140	rs2745426
27	rs13464	65	rs4240467	103	rs7226305	141	rs6854081
28	rs11675051	66	rs7851693	104	rs7217932	142	rs2228570
29	rs12995369	67	rs3905706	105	rs4796995	143	rs7975232
30	rs6436440	68	rs13734	106	rs884205	144	rs1544410
31	rs10510373	69	rs7071206	107	rs2717096	145	rs731236
32	rs2291296	70	rs2784767	108	rs7257450	146	rs1107946
33	rs7427438	71	rs7084921	109	rs10416218	147	rs2412298
34	rs430727	72	rs11602954	110	rs3790160	148	rs180012
35	rs1026364	73	rs7108738	111	rs4817775	149	rs545382
36	rs344081	74	rs10835187	112	rs4820539	150	rs2277268
37	rs3755955	75	rs163879	113	rs5934507	151	rs9340799
38	rs4832734	76	rs7932354	114	rs5926033	152	rs2234693

На втором этапе проведена подготовка данных для машинного обучения. Для этого создан актуальный датасет в виде таблицы в формате xlsx. В таблице представлено 1202 образца обезличенных данных. То есть номеру строки/образца соответствует идентификатор определенного пациента.

На третьем этапе построены модели CatBoostClassifier в составе платформы CatBoost (использует метод повышения градиента с итеративной постройкой множества деревьев решений, когда каждое последующее дерево улучшает результат предыдущего, что приводит к лучшим результатам) и нейросетевая модель на основе полносвязных слоев (Dense). Проведенные эксперименты условно можно разделить на две категории.

На четвертом этапе после обучения моделей CatBoostClassifier для обеих групп пациентов была проведена кросс-валидация для оценки качества работы модели, помогающая сравнить между собой различные модели и выбрать наилучшую для прогнозирования первичного остеопороза в исследованных выборках мужчин и женщин.

## Описание медицинского вмешательства

Уровень МПКТ измеряли методом двухфазной абсорбционной рентгеновской денситометрии (DEXA) с использованием системы Hologic QDR 4500/A DXA (США) в стандартных локализациях (шейка бедра и поясничный отдел позвоночника). Согласно критериям Всемирной организации здравоохранения общая выборка была разделена в соответствии с T-критерием — значения от +2,5 до -0,9 стандартных отклонений (SD) — нормальная МПКТ, меньше -1 и больше -2,5 SD — остеопения, остеопороз — от -2,5 SD и ниже (≤2,5 SD) [13–14].

## Методы

Подготовка данных для машинного обучения

В качестве целевой переменной выбиралась колонка «диагноз», которая имеет два значения: нормальный уровень МПКТ (нет риска), низкий уровень МПКТ (высокий риск). Датасет содержит 4 численных переменных: возраст, вес, рост, индекс массы тела, а также 152 локуса (табл. 2), пол и наличие случаев перелома у пациента. Каждый локус характеризуется набором пар нуклеотидов в таблице в виде сочетания двух латинских букв. Датасет содержит уникальные сочетания пар нуклеотидов (при последующем перекодировании в One Hot Encoding (OHE) учтены все возможные сочетания, с учетом потенциального наполнения датасета дополнительными локусами с расширенным набором пар нуклеотидов в будущем. Пол закодирован как 0 — мужчины; 1 — женщины. Перелом (Fracture) закодирован как 0 — отсутствие перелома; 1 — наличие как минимум одного перелома. Файл исходных данных сначала конвертировался из формата «xlsx» в «csv» и затем подавался на анализ и предобработку в рабочую среду. Ввиду полового диморфизма в этиопатогенезе остеопороза, различий в эндокринной регуляции метаболизма костной ткани у мужчин и женщин, исходный датасет был разделен на два по половому признаку для применения в обучении двух разных моделей. При этом пол исключается из набора переменных для дальнейшей обработки. Особое внимание уделено проверке отсутствия пропусков данных, симметричности выборок по категориям, наличию корреляции между переменными в датасете и применению соответствующих методов компенсации. Категориальные переменные (включая метки) преобразовывались в вид OHE либо применяемой платформой МО, либо с помощью соответствующей программной функции в случае обучения нейросети. Численные переменные подвергались масштабированию в диапазон чисел от 0 до 1. В машинные модели обучения в качестве зависимой и независимой переменной был включен показатель «Низкий уровень МПКТ», который объединял пациентов с остеопорозом и остеопенией. Подготовка данных, обучение модели, проверка и тестирование проводились в среде Google Colab на платформе с открытым исходным кодом TensorFlow, а также на платформе CatBoost. В качестве высокоуровневого интерфейса прикладного программирования для TensorFlow применялся API Keras. Язык программирования — Python 3. Основные модули, примененные в работе, включают в себя pandas (работа с табличными данными), numpy (работа с массивами), scikit-learn (подготовка данных и формирование матрицы ошибок), matplotlib (отображение графиков), Plotly (динамическое отображение графиков), SweetVIZ (для первичного анализа). Далее в результатах будут использоваться термины «тестовая выборка» и «проверочная выборка».

## Этическая экспертиза

Все участники подписали информированное согласие на включение в исследование в соответствии со стандартами, разработанными Хельсинкской декларацией Всемирной медицинской ассоциации «Этические принципы проведения научных медицинских исследований с участием людей в качестве субъектов исследования» и с одобрения локального биоэтического комитета Института биохимии и генетики УФИЦ РАН (протокол №14 от 15.09.2016).

## РЕЗУЛЬТАТЫ

Для обеих групп экспериментов оценивались два подхода — обработка данных с помощью модели на основе полносвязных нейронных слоев (Dense) и модель CatBoostClassifier. При этом использование CatBoostClassifier дало более точные результаты (средняя точность классификации 64,5% для мужчин и 73% для женщин) на независимом датасете и выглядит несколько проще, так как метод выполняет предварительную обработку данных как часть алгоритма. В рамках данной статьи мы решили сосредоточиться на описании методики и результатов предсказания низкого уровня МПКТ.

Для второй группы экспериментов проводилось разделение на 2 класса (нормальный уровень МПКТ (нет риска)/низкий уровень МПКТ (высокий риск)). Такой подход выглядит оправданным при первичной оценке эффективности метода в силу простоты и малого размера исходной базы данных. Кроме того, модели, строящиеся на малом наборе данных, склонны к переобучению, и, чтобы избежать эффекта переобучения, применялся метод раннего прекращения обучения моделей. Для повышения стабильности результатов при обучении модели использовалось перемешивание данных и соблюдение пропорций при разделении исходного набора данных на обучающий и тестовый, а также контроль весов классов при делении данных на мини-пакеты.

Особенностью нашего исследования является то, что в моделях прогнозирования последовательно, методом включения и исключения из изначально большого набора данных, была сформирована отдельная модель из небольшого числа значимых маркеров, которые, по всей видимости, являются значимыми предикторами в модели машинного обучения. Это может обосновываться тем, что модели прогнозирования лучше всего предсказывают низкий уровень МПКТ именно при включении качественно значимых предикторов, в то время как при анализе крупных массивов снижается качество обучения моделей из-за наличия рисковых аллелей с низким эффектом. В частности, мы начали с исследования результатов работы моделей для 152 ДНК-маркеров без включения других клинически значимых показателей, например, рост и вес пациентов. Влияние каждой из переменных на результат оказалось слишком «шумным», т.е. простые модели не смогли выделить закономерности, связывающие всю совокупность комбинаций нуклеотидов в изначально выбранной широкой группе локусов с высокой вероятностью низкого уровня МПКТ и перелома, показатели чувствительности и специфичности моделей при включении всех ДНК-локусов не превышали 60%. Анализируя различные варианты моделей, мы установили, что наиболее высокой эффективности (составляющей AUC = 0,81 для мужчин и AUC = 0,82 для женщин на независимом датасете) достигает модель прогнозирования низкого уровня минеральной плотности костной ткани, в которую вошли 6 полиморфных вариантов гена остеопротегерина (OPG) (rs2073618, rs2073617, rs7844539, rs3102735, rs3134069) и 5 полиморфных вариантов сайтов связывания микроРНК в мРНК таргетных генов, участвующих в костном метаболизме (COL11A1 — rs1031820, FGF2 — rs6854081, miR-146 — rs2910164, ZNF239 — rs10793442, SPARC — rs1054204 и VDR — rs11540149).

Далее в каждой из выборок мужчин и женщин исследуeмые предикторы были использованы для обучения нейросети с дальнейшей кросс-валидацией.

## Нейросетевой анализ датасета выборки мужчин

Процесс и результаты обучения представлены на рисунках 1–3.

**Figure:**
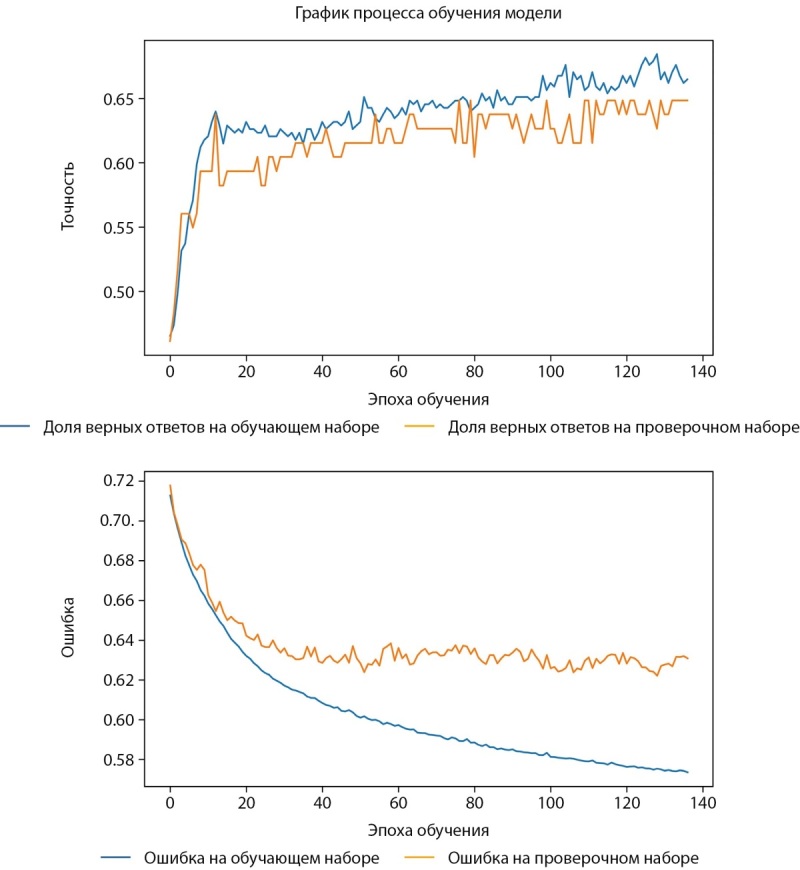
Рисунок 1. График процесса обучения (слева — точность, справа — ошибка).

**Figure:**
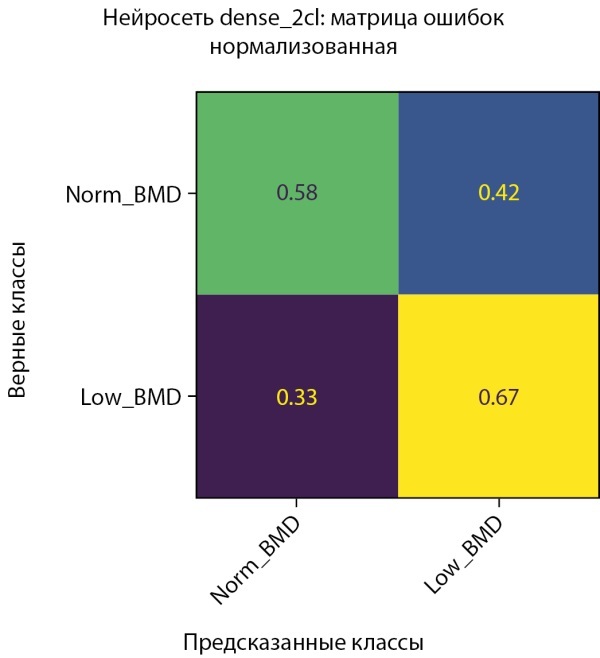
Рисунок 2. Нормализованная матрица ошибок предсказания двух классов МПКТ на проверочной выборке (мужчины).

**Figure:**
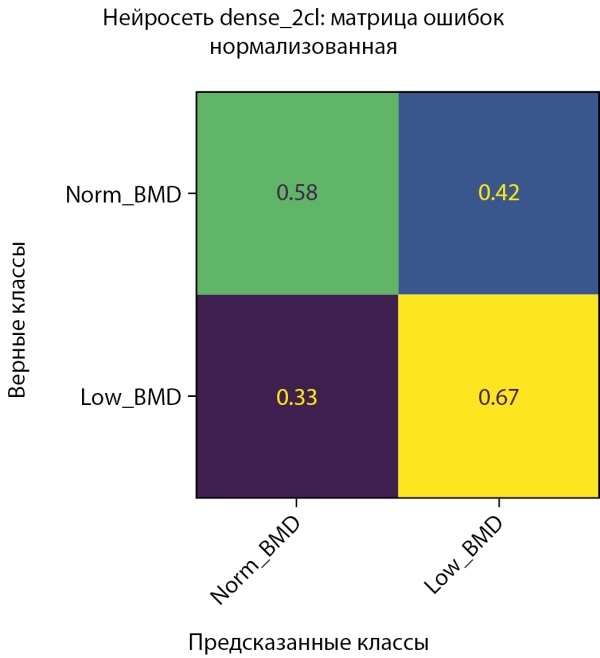
Рисунок 3. Нормализованная матрица ошибок предсказания двух классов МПКТ на тестовой выборке (мужчины).

Средняя точность классификации по 2-м классам достигла 62,5%. При этом класс пониженного уровня МПКТ определяется лучше, чем нормальный уровень как на проверочной части датасета, так и на тестовой. Истинно-положительный результат (True-Positive) для нормального уровня МПКТ был зафиксирован лишь в 58% случаев, тогда как для пониженного уровня МПКТ — в 67% случаев.

Для оценки качества бинарной классификации применен ROC-анализ (рис. 4). ROC-кривая отображает соотношение между долей истинно-положительных классификаций (True Positive Rate), называемой чувствительностью алгоритма классификации, и долей ложно-положительных классификаций. Показатель AUC (площадь под кривой) у мужчин достиг 0,68 (рис. 4).

**Figure:**
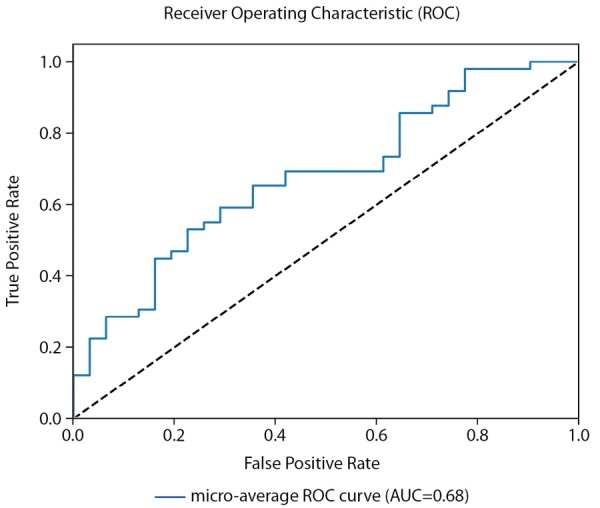
Рисунок 4. ROC-кривая, построенная по предсказаниям тестовой выборки (мужчины).

## Нейросетевой анализ датасета выборки женщин

Процесс обучения нейросети в выборке женщин представлены на рисунке 5.

**Figure:**
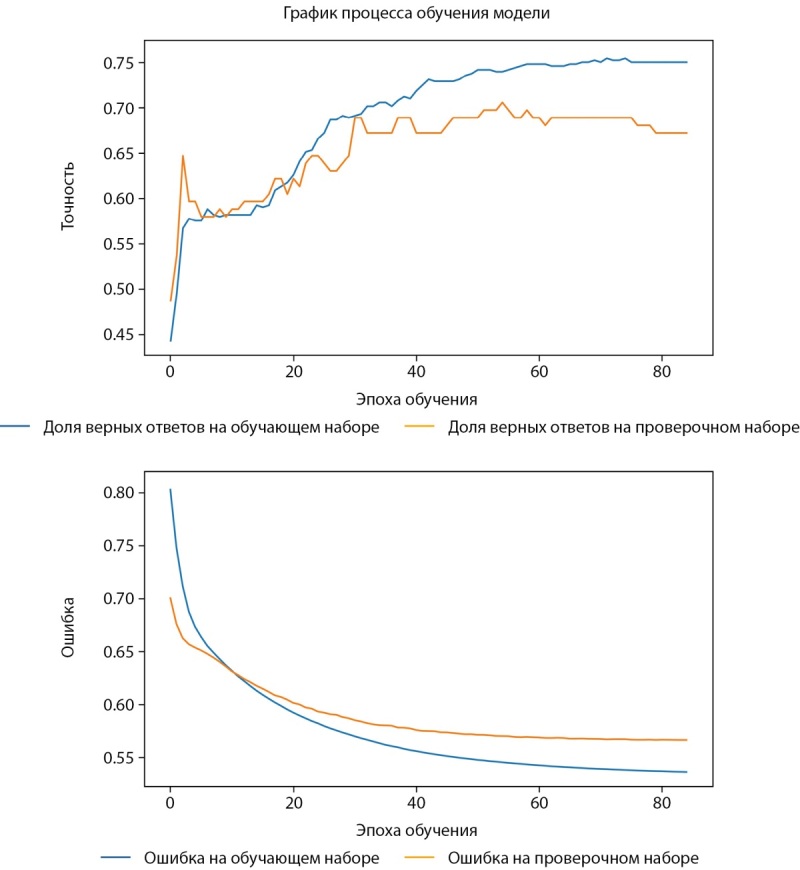
Рисунок 5. График процесса обучения.

Средняя точность классификации по 2-м классам достигла 73%. При этом класс пониженного уровня МПКТ определяется несколько лучше, чем нормальный уровень как на проверочной части датасета, так и на тестовой. Однако разброс в точности определения классов ниже, чем на датасете мужчин (4% против 9%). Предположительно, это объясняется большей корреляцией параметров датасета с диагнозом и большей выборкой датасета женщин. Истинно-положительный результат (True Positive) для нормального уровня МПКТ был зафиксирован лишь в 71% случаев, тогда как для пониженного уровня МПКТ — в 75%. ROC-кривая для модели, построенной для датасета женщин, заметно ближе к идеальной чем для модели, построенной для датасета мужчин (AUC=0,78 против 0,68) (рис. 6).

**Figure:**
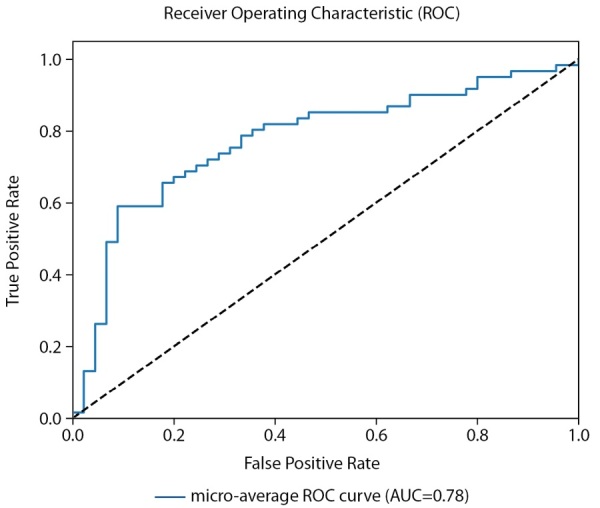
Рисунок 6. ROC-кривая, построенная по предсказаниям тестовой выборки (женщины).

## Применение инструмента CatBoost на датасете выборки мужчин

Обучение модели на датасете мужчин с помощью модели CatBoostClassifier показало наилучшие результаты с большинством базовых гиперпараметров. Как и при обучении нейросети, точность и стабильность результатов предсказания оказались хуже, чем в модели, построенной на датасете женщин. При обучении также использовался метод ранней остановки обучения, чтобы избежать переобучения (рис. 7–8).

**Figure:**
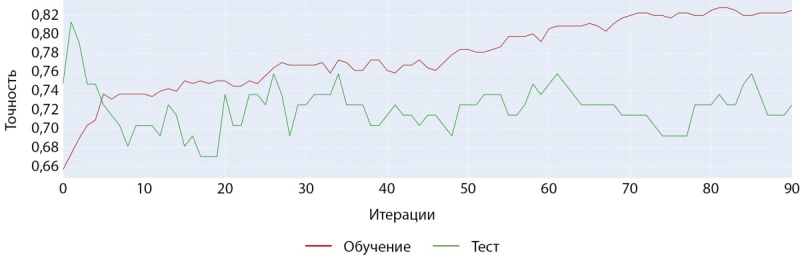
Рисунок 7. График точности при обучении модели CatBoostClassifier (мужчины).

**Figure:**
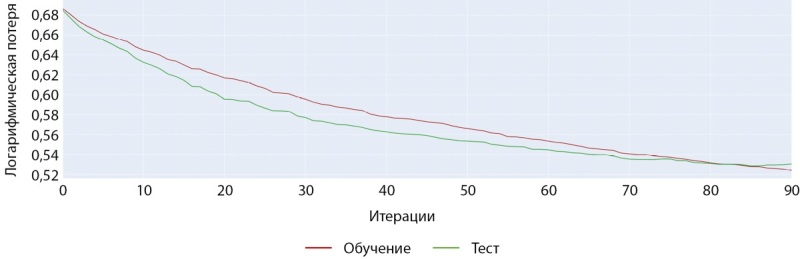
Рисунок 8. График ошибки при обучении модели CatBoostClassifier (мужчины).

На рисунке 8 по оси «ординат» отображена метрика функции «логарифмическая потеря», которая отражает логарифмическую вероятность истинных меток с учетом прогнозируемых вероятностей. Более низкие значения указывают на лучшую производительность, при этом 0 соответствует идеальной модели. Эта метрика используется для оптимизации модели во время обучения и оценки ее точности.

С помощью модели CatBoostClassifier нам удалось увеличить среднюю точность предсказания на тестовом наборе данных на 2% (64,5% против 62,5%). Однако, как показывает разница результатов в матрицах ошибок, построенных на проверочной и тестовой выборках (ср. рисунки 9–10), текущие данные слишком неоднородны, что приводит к более значимой вариативности точности предсказаний диагноза у мужчин. Это же подтверждается и результатами кросс-валидации (ср. рисунок 19–20).

**Figure:**
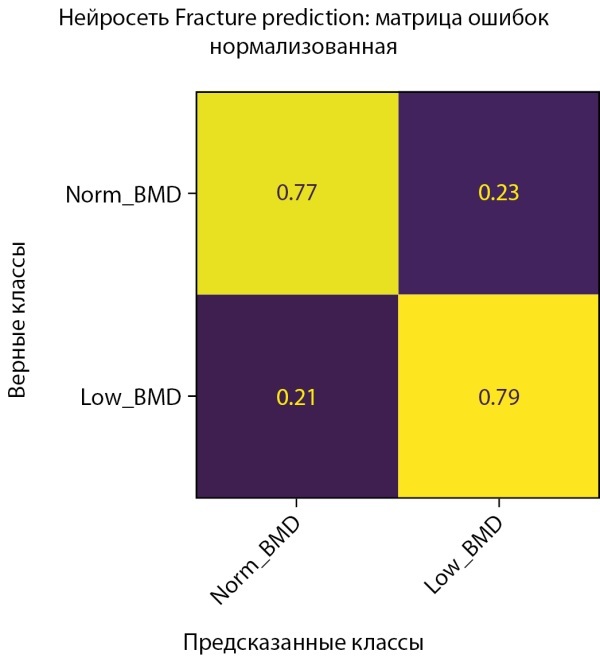
Рисунок 9. Нормализованная матрица ошибок предсказания двух классов МПКТ на проверочной выборке (мужчины).

**Figure:**
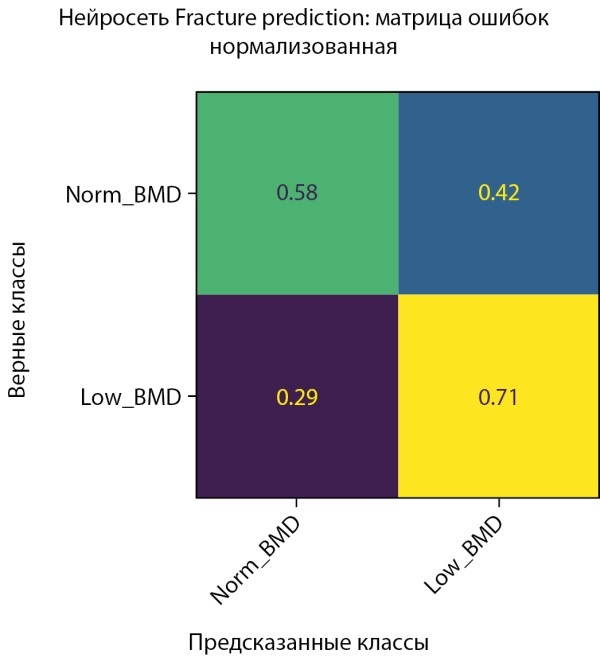
Рисунок 10. Нормализованная матрица ошибок предсказания двух классов МПКТ на тестовой выборке (мужчины).

В то же время наблюдается заметное увеличение качества модели (AUC=0,81 против 0,64) (рис. 11).

**Figure:**
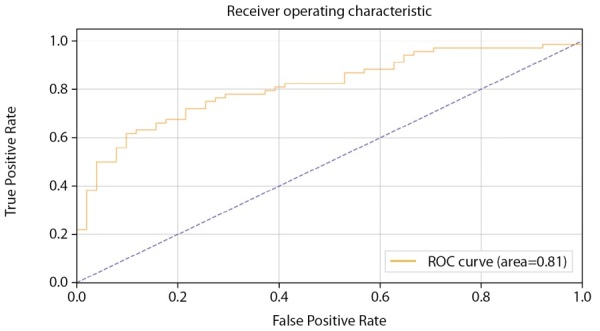
Рисунок 11. ROC-кривая, построенная по предсказаниям тестовой выборки (мужчины).

Модель CatBoostClassifier имеет встроенный функционал, помогающий определить желаемый порог решающего правила бинарной классификации. График на рисунке 12 построен на данных проверочного набора. Проекция точки пересечения графиков функций FPR и FNR на ось Threshold (порог) дает рекомендуемый оптимальный порог решающего правила, который может быть намеренно смещен в ту или иную сторону (обычно в сторону большей вероятности определения заболевания).

Специфика такого смещения довольно часто используется при ранней первичной диагностке.

**Figure:**
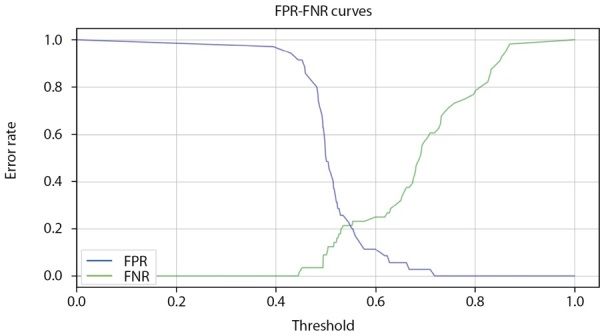
Рисунок 12. Определение порога бинарной классификации в модели CatBoostClassifier по пересечению кривых FPR и FNR (мужчины).

## Применение инструмента CatBoost на датасете выборки женщин

Обучение модели на датасете женщин с помощью модели CatBoostClassifier также показало наилучшие результаты с большинством базовых гиперпараметров. Как видно на рисунках 13 и 14 обучение на данном наборе шло более успешно и достигло более значимых показателей точности.

**Figure:**
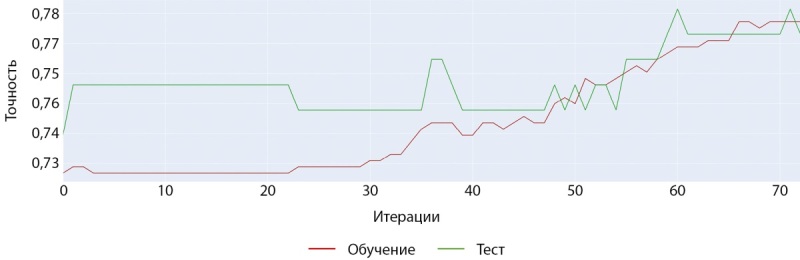
Рисунок 13. График точности при обучении модели CatBoostClassifier (женщины).

**Figure:**
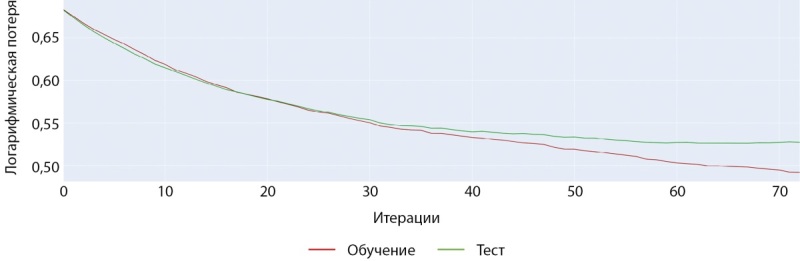
Рисунок 14. График ошибки при обучении модели CatBoostClassifier (женщины).

Обучение было остановлено в момент достижения минимума ошибки, и параметры модели были сохранены именно в этом состоянии. На рисунке 15 показана полная симметричность истинно-положительных и ложноположительных результатов. Это достигнуто благодаря выбору рекомендованного порога решающего правила, рассчитанного на проверочных данных. На данный момент в датасете женщин также присутствует некоторая неоднородность, которая проявляется в несимметричности истинно-положительных и ложноположительных результатов, полученных на тестовой выборке (рис. 16).

**Figure:**
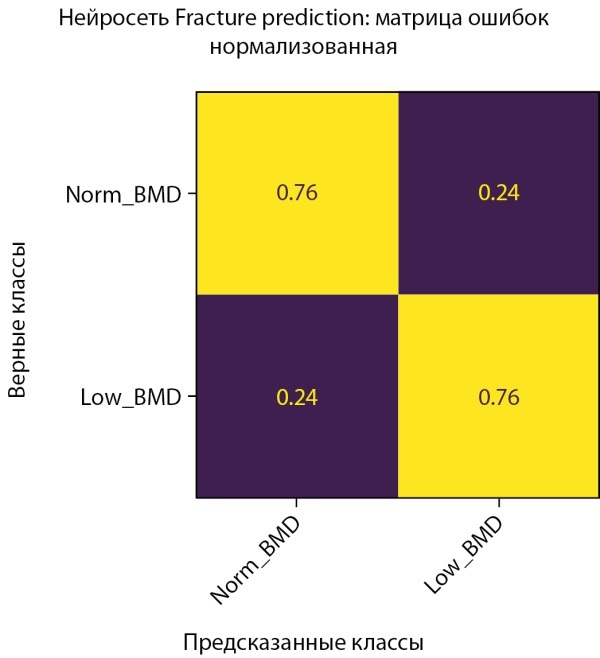
Рисунок 15. Нормализованная матрица ошибок предсказания двух классов МПКТ на проверочной выборке (женщины).

**Figure:**
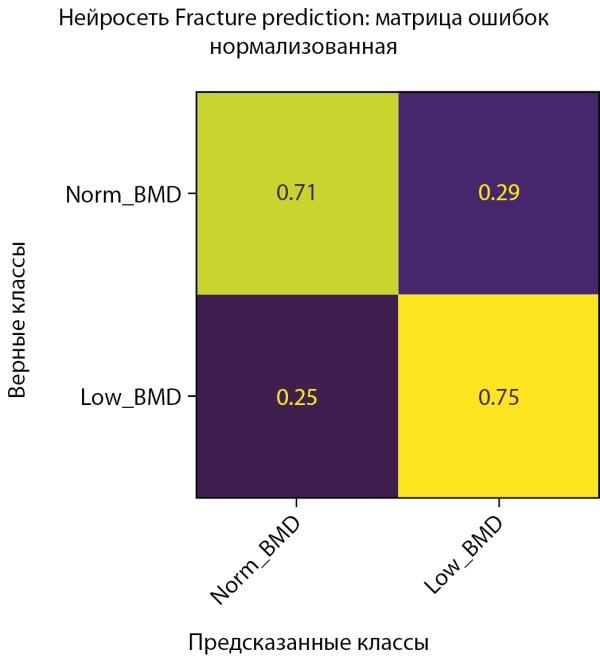
Рисунок 16. Нормализованная матрица ошибок предсказания двух классов МПКТ на тестовой выборке (женщины).

Показатель качества модели AUC в модели CatBoostClassifier на датасете женщин также получил улучшение по сравнению с моделью на основе нейросети (AUC=0,82 против 0,78) (рис. 17–18).

**Figure:**
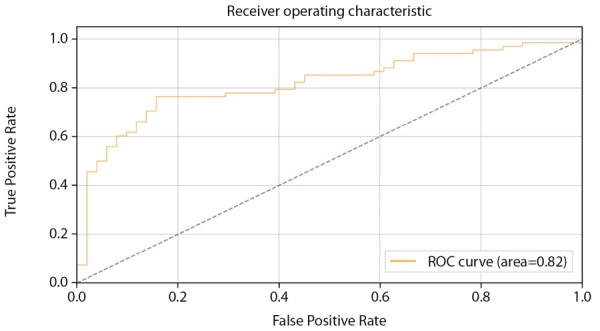
Рисунок 17. ROC-кривая, построенная по предсказаниям тестовой выборки (женщины).

**Figure:**
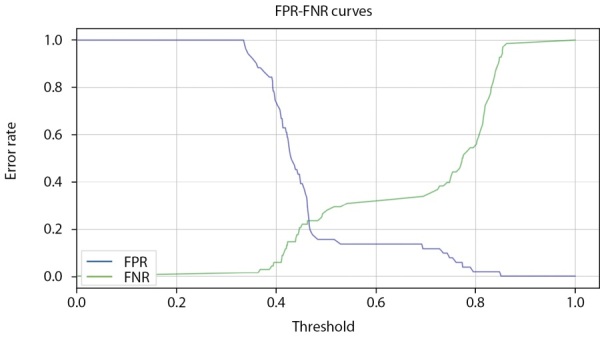
Рисунок 18. Определение порога бинарной классификации в модели CatBoostClassifier по пересечению кривых FPR и FNR (женщины).

## Кросс-валидация полученных результатов

В данной работе мы провели оценку работы двух моделей, одной модели для мужчин и одной модели для женщин, чтобы убедиться в качестве работы этих моделей с разными данными разделив исходный датасет на 5 частей встроенными средствами платформы CatBoost. Кривая на рисунках 19 и 20 на фоне закрашенной области показывает среднее значение точности модели для датасетов, а сама закрашенная область характеризует девиацию точности при кросс-валидации.

**Figure:**
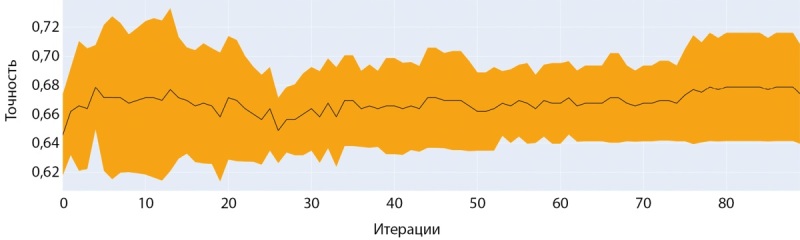
Рисунок 19. Точность на тестовых наборах при пятикратной кросс-валидации (мужчины).

**Figure:**
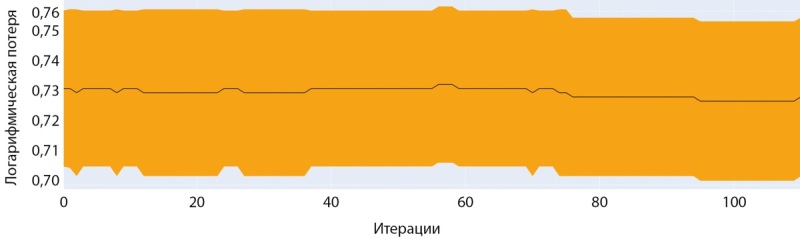
Рисунок 20. Точность на тестовых наборах при пятикратной кросс-валидации (женщины).

Аналогично кривые на рисунке 20 на фоне закрашенной области показывают среднее значение ошибки (Loss) модели для датасетов, а сама закрашенная область характеризует девиацию ошибки при кросс-валидации.

Как видно из сравнения графиков, модель для датасета женщин показывает большую стабильность и меньшую вариативность предсказаний диагноза. Отчасти это объясняется разницей в размерах датасетов. В любом случае, модель для женщин ближе к возможности практического применения для диагностики.

## ОБСУЖДЕНИЕ

В настоящем исследовании впервые в России применены подходы машинного обучения, в том числе нейросетевого анализа, для разработки модели прогнозирования риска развития остеопороза. Выявленные в ходе работы модели прогнозирования и оценка диагностической эффективности методом МО показали, что для поставленной цели достаточно нескольких слоев полносвязной архитектуры НС или применения алгоритмов МО, таких как CatBoostClassifier с правильно подобранными гиперпараметрами и функциями активации. Проведенное исследование подтвердило перспективность применения новых методов обработки данных на основе МО и НС, а также определило дальнейшие направления работы для увеличения точности диагностирования остеопороза в зависимости от генетической предрасположенности и ряда других характеристик. ДНК-маркеры риска, идентифицированные в GWAS и метаанализах, представляют собой проблему в традиционном статистическом анализе, поскольку величина их эффекта риска очень мала. Каждый ассоциированный с остеопорозом однонуклеотидный полиморфизм вносит минимальный вклад в вариабельность уровня минеральной плотности костной ткани (МПКТ), поэтому использование машинного обучения представляет высокую ценность как инструмент, позволяющий учитывать множество предикторов с малым эффектом.

## Репрезентативность выборок

Исследование выполнено на репрезентативной выборке, позволяющей получить статистически релевантные результаты.

## Сопоставление с другими публикациями

Хорошо зарекомендовавшие себя методы линейной и логистической регрессии до сих пор используются при моделировании прогноза риска переломов, однако модели машинного обучения также нашли свое применение при изучении остеопороза и могут предложить более перспективные системы прогнозирования риска развития ОП. Так, научной группе Ho-Le с соавт. (2017 г.) удалось добиться значимого результата, разработав чувствительные модели прогнозированию риска переломов шейки бедра на основе нейросетевого анализа (с показателем AUC до 0,7–0,85) [15]. Ferizi с соавт. (2019 г.) использовали данные магнитно-резонансной томографии (МРТ) 32 женщин с предшествующими переломами и, сравнив 15 так называемых классификаторов машинного обучения, пришли к выводу, что логистическая регрессия, линейный дискриминант или алгоритм «усиленных деревьев принятия решений» обеспечивают наилучший баланс чувствительности и специфичности для прогнозирования остеопоротических переломов, а также то, что инструмент прогнозирования переломов FRAX может быть улучшен только технологией машинного обучения [16]. Интересным представляется исследование Wu Q. с соавт. (2021 г.), которые применили подходы МО для прогнозирования уровня МПКТ с использованием генетических (1103 SNP) и фенотипических данных пожилых мужчин (N=5130). Они установили, что модели МО проявили себя гораздо эффективнее линейной регрессии с регуляризацией лассо, а метод «повышения градиента» показал лучшие результаты. Наибольшей прогностической силы их модель достигла, когда вклад SNP-маркеров учитывался дискретно без поправки на коэффициент полигенного риска, однако это создало помехи при оценке общего вклада генетических предикторов [17].

Тем не менее полигенную оценку риска (PRS) следует относить к наиболее передовым подходам при разработке прогностических моделей, ввиду того, что эффекты риска (OR/beta) могут быть объединены в полигенную шкалу риска, отражающую часть индивидуальной восприимчивости человека к остеопорозу с учетом специфики генофонда изучаемой популяции [18–19]. Ранее, на основе PRS анализа, мы разработали модели прогнозирования риска переломов у женщин в постменопаузе из Волго-Уральского региона России с эффективностью 74% (AUC=0,740; OR=2,9 (95% ДИ 2,353–3,536)), формирования низкого уровня МПКТ с эффективностью 79% (AUC=0,790; OR=3,94 (95% ДИ 2,993–5,337)), а также риска переломов в сочетании с низким уровнем МПКТ с точностью 85% (AUC=0,850; OR=6,6 (95%ДИ 4,411–10,608)) [11]. Интересным представляются результаты работы Chen с соавт. (2021 г.), которые провели анализ профиля экспрессии генов с использованием машинного обучения, изучили модель из 176 генов, которые, по прогнозам, были ассоциированы с возникновением ОП. Всего были идентифицированы 50 ключевых генов. Затем из них 22 гена были проверены на основе поэтапного регрессионного анализа, после чего 9 генов были дополнительно проверены с использованием многомерного регрессионного анализа с порогом значимости p<0,01. На этой основе ими была разработана модель с высокой предсказательной ценностью на основе значимых полиморфных локусов 9 генов: LCK (протоонкоген LCK, тирозинкиназа семейства Src.), LY9 (антиген лимфоцитов 9), CD5 (молекула CD5), P2RY8 (член семейства 8-рецепторов P2Y), KCTD7 (Домен тетрамеризации калиевого канала, содержащий 7), MDN1 (Домен тетрамеризации калиевого канала, содержащий 7), ITK (киназа Т-клеток, индуцируемая IL2), CAPN2 (кальпаин 2) и HTT (гентингтин) [20]. Однако результаты не были воспроизведены.

## Направления дальнейших исследований

Несмотря на то, что подход машинного обучения улучшает способность прогнозирования, метод требует дальнейшей апробации на расширенной выборке с использованием большего охвата анализируемых параметров. Прогностические модели риска остеопороза требуют дальнейшего совершенствования. Для современной предиктивной медицины внедрение методов МО является приоритетной задачей. Это объясняется ростом распространенности социально значимых заболеваний в мире, а это, в свою очередь, ложится огромным экономическим бременем на государство при финансировании реабилитационных центров и других структур здравоохранения, ответственных за лечение пациентов с остеопоретическими переломами. На сегодняшний день лишь несколько моделей МО прогнозируют остеопоротические переломы и улучшают прогнозирование переломов бедра за пределами логистической регрессии [21]. Также алгоритмы МО применены для определения вероятности переломов бедра, продолжительности реабилитации и продолжительности последующего ухода за пациентами с переломом бедра [22]. Необходимо проведение сравнительного анализа и проверки эффективности машинного обучения нейросетей в способности улучшать модели прогнозирования риска развития остеопороза на основе клинико-генетических данных. Машинное обучение направлено на реализацию программных алгоритмов, способных довести точность прогнозирования того или иного исхода на основе сложных данных до 70–80%, кроме того, для этого метода характерна способность моделировать сложные взаимосвязи большого количества переменных. Повышение градиента, метод «случайного леса» и сверточная нейронная сеть — широко используемые подходы МО для моделирования сложных медицинских данных [23]. Однако эффективность этих моделей машинного обучения для прогнозирования риска переломов остается в значительной степени неизвестной.

## Значимость результатов

Основываясь на наших предыдущих научных исследованиях, мы выделили наиболее значимые для прогнозирования вероятности низкого уровня МПКТ ДНК-маркеры и клинически значимые характеристики, которые в совокупности повышают возможности даже простых моделей выполнять медицинский скрининг, помогая первичному звену медицинской помощи принимать квалифицированное решение о необходимости направить пациента на дополнительные и дорогостоящие исследования. Однако в то же время проект является пилотным с точки зрения исследования остеопороза с использованием клинико-генетических данных на основе технологий нейросетевого анализа, поэтому расширение выборки для усиления потенциала обучения моделей и включение дополнительных ДНК-маркеров будет продолжено.

На сегодняшний день существуют две основные проблемы внедрения технологии машинного обучения в область диагностической медицины для прогнозирования риска остеопоротических переломов.

Для решения этих проблем существует 2 пути:

1) поиск новых алгоритмов анализа массивов генетической информации, в том числе с использованием глубокого машинного обучения и современных нейросетевых моделей, которые позволили бы учесть больше ДНК-маркеров без потери качества и прогностической мощности моделей;

2) обучение нейросетевых моделей на полногеномных данных из выборок пациентов с первичным остеопорозом, проживающих на территории России, для учета специфики генофонда российских популяций.

Мы установили, что метод классификации на основе алгоритма «решающих деревьев» опережает решение на основе нейронной сети, но мы знаем, что с накоплением большего количества данных нейронная сеть будет работать не хуже, благодаря расширению потенциала обучения. Важно помнить, что важным преимуществом перед классическими статистическими методами является то, что методы МО способны обрабатывать данные большого размера и включать различные нелинейные взаимодействия между генетическими вариантами/предикторами, которые не могут быть выявлены традиционными методами моделирования. В данной работе удалось добиться лучших результатов используя метод классификации на основе алгоритма «решающих деревьев». Мы надеемся, что в результате дальнейшей работы могут быть получены более точные результаты и с применением методов на основе нейросетей. Таким образом, подходы МО обладают большим потенциалом для улучшения прогнозирования риска переломов и уровня МПКТ и способны служить подспорьем для прикладных разработок в медицине.

## ЗАКЛЮЧЕНИЕ

Таким образом, в результате проведенного исследования мы разработали прогностическую модель на основе CatBoostClassifier и модель на основе полносвязных слоев нейросетей, предсказывающие риск формирования низкого уровня минеральной плотности костной ткани с учетом клинико-генетических данных с эффективностью, достигающей AUC=0,81 для мужчин и AUC=0,82 для женщин на независимом датасете. Метод машинного обучения следует рассматривать как инструмент для обеспечения лучшей стратификации риска ОП для выявления лиц с высоким риском перелома, особенно с учетом того, что генетические данные являются значимым предикторами первичного остеопороза.

## ДОПОЛНИТЕЛЬНАЯ ИНФОРМАЦИЯ

Источники финансирования. Работа выполнена при поддержке Министерства науки и высшего образования Российской Федерации (соглашение № 075-15-2022-310 от 20 апреля 2022 г.).

Конфликт интересов. Авторы заявляют об отсутствии конфликта интересов.

Участие авторов. Ялаев Б.И. — написание статьи, обобщение и интерпретация результатов с сопутствующим анализом зарубежной литературы по теме исследования, внесение в рукопись существенной правки с целью повышения научной ценности статьи; Новиков А.В. — оптимизация алгоритмов машинного обучения, подбор программных протоколов для улучшения прогностической ценности моделей; Минниахметов И.Р. — администрирование проекта, редактирование статьи; Хусаинова Р.И. — руководство, концептуализация и администрирование проекта, редактирование статьи.
